# Dr. Joseph Clark

**Published:** 1918-09

**Authors:** 


**Dr. Joseph Clark,** Olean, died July 22 in Buffalo, age 69. He graduated from the New York University in 1885.

